# Corolla retention after pollination facilitates the development of fertilized ovules in *Fritillaria delavayi* (Liliaceae)

**DOI:** 10.1038/s41598-018-37358-0

**Published:** 2019-01-24

**Authors:** Yongqian Gao, Changming Wang, Bo Song, Fan Du

**Affiliations:** 1 College of Biodiversity Conservation and Utilization, Southwest Forestry University, 300 Bailong Road, Kunming, 650224 Yunnan P. R. China; 2 Yunnan Forestry Technological College, 1 Jindian, Kunming, 650224 Yunnan P. R. China; 3 Faculty of Forestry, Southwest Forestry University, 300 Bailong Road, Kunming, 650224 Yunnan P. R. China; 4 Key Laboratory for Plant Diversity and Biogeography of East Asian, Kunming Institute of Botany, Chinese Academy of Sciences, 132 Lanhei Road, Kunming, 650201 Yunnan P. R. China

## Abstract

Corollas (or perianths), considered to contribute to pollinator attraction during anthesis, persist after anthesis in many plants. However, their post-floral function has been little investigated within a cost-benefit framework. We explored the adaptive significance of corolla retention after anthesis for reproduction in *Fritillaria delavayi*, a perennial herb endemic to the alpine areas of the Hengduan Mountains, southwestern China. We examined whether the persistent corollas enhance reproductive success during seed development. Persistent corollas increased fruit temperature on sunny days, and greatly decreased the intensity of ultraviolet-B/C (UV-B/C) radiation reaching fruits. When corollas were removed immediately after pollination, fecundity and progeny quality were adversely affected. Measurements of flower mass and size showed no further corolla growth during fruiting, and respiration and transpiration tests demonstrated that both respiration rate and transpiration rate of corollas were much lower during fruiting than during flowering, indicating a slight additional resource investment in corolla retention after anthesis. Thus, seed production by *F. delavayi* may be facilitated by corolla retention during seed development at only a small physiological cost. We conclude that corolla retention may be an adaptive strategy that enhances female reproductive success by having a protective role for ripening seeds in the harsh conditions at high elevation.

## Introduction

In order to ensure reproductive success, flowering plants exhibit an astonishing diversity of floral traits; these include different colors of petals^1,2^, variable flower orientation^3,4^, individual flower movement^5,6^, and extrafloral structures^7,8^. In particular, the variability of corollas or perianths is associated with an impressive variety of reproductive strategies^5,9,10^. It is well established that the primary function of corollas is to attract pollinators^2^. For example, corolla color or size can influence pollinator attraction, with bright or large flowers attracting more pollinators than dark or small flowers^11,12^. In addition to the advertisement effects, corolla shape^9^ and movement^10^ can affect the behavior of visiting insects to enhance the pollination success. For example, bowl-shaped flowers can focus sunlight in a way similar to a parabolic reflector, resulting in a heating of the interior^9^, contributing to pollinator attraction because warm flowers are preferred by insect pollinators^5,13^. In addition, corolla orientation can enhance pollen viability. Wang *et al*.^3^ found that a pendulous corolla in *Anisodus luridus* from the Qinghai-Tibet Plateau can protect pollen grains in anthers and on stigmas from rain wash and intense solar radiation.

In fact, in many plant species, corollas persist well beyond the completion of pollination, usually until fruit maturation and seed dispersal, suggesting that they have an additional function during seed ripening unrelated to the pollination success. However, compared to the considerable attention that has been paid to the functions of corollas during flowering, investigations into their post-floral functions are strikingly scarce^14^ and the few studies available have mostly focused on the function of the calyx^15^. Furthermore, these studies have not always confirmed any adaptive value of perianth persistence. In some cases, perianth persistence has been found to have beneficial effects on plant fitness by contributing photosynthates to developing fruits and seeds, heating the developing fruits, or protecting seeds from seed predation^2,14,16^. However, some studies report that the persistent calyx has no effect on the development of fruits^17,18^, or even decreases plant fitness because it provides larvae of frugivores with a refuge from parasitism, resulting in a higher percentage of fruits being eaten by larvae^19^. In addition, corolla retention often incurs physiological costs, such as investment in biomass, respiration and water loss by evaporation, which may negatively affect fruit and seed production^20–22^. Consequently, further studies of a variety of species, within a cost-benefit framework and in various environments, are needed to assess the adaptive significance of corolla retention during seed development.

**Figure 1:**
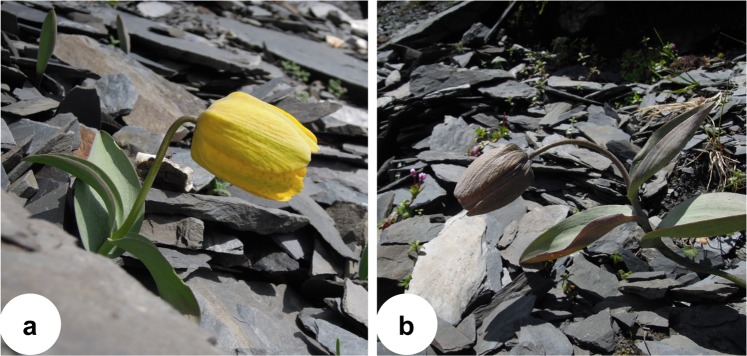
*Fritillaria delavayi* during flowering (**a**) and fruiting (**b**). The color of tepals changes from bright yellow to gray and they encloses fruits during seed development.

The alpine zone of the Hengduan Mountains region in Southwest China is generally characterized by low air temperatures, frequent rain and high air humidity, short periods of intense solar radiation and a short growing season^23^. These weather conditions and the short growing season are considered important factors that limit plant reproduction^24^. Plants in such habitats have developed a variety of adaptive mechanisms to cope with the hostile environmental conditions^24,25^. The bell-shaped, pendulous corollas of *Fritillaria delavayi*, a perennial herb endemic to the alpine zone of the Hengduan Mountains, are retained after flowering and tepal color changes from bright yellow to gray (Fig. 1), suggesting the existence of additional functions of the retained corolla during seed development. In this study, we aimed to determine the various functions of corolla retention in *F. delavayi* after anthesis. As dark flowers have been found to absorb solar radiation efficiently^26^, in combination with the high effectiveness of corollas or perianths in blocking UV radiation which has been documented in many plant species^3,27,28^, we hypothesized that the dark, closed corolla of *F. delavayi* may facilitate the development of fertilized ovules by increasing interior temperature and forming a protective barrier against ultraviolet radiation. To test this hypothesis, we examined (1) the effect of corolla retention on interior temperature and intensity of UV-B/C radiation; (2) the possible costs of corolla retention in terms of growth (increase in biomass), respiration and transpiration; (3) the effect of corolla retention during seed ripening on female fitness.

## Results

### Effects of corolla retention on interior microenvironment

#### Temperature

**Figure 2:**
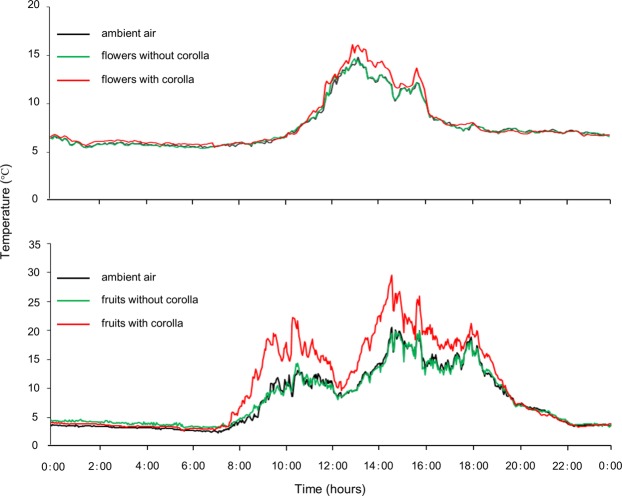
Variation in temperature in three different treatments during flowering (**a**, rain started at 13:40 h on 8-Jul-2016) and fruiting (**b**, rain started at 10:30 h on 7-Aug-2016) (n = 3).

During the measurement period, surface temperatures of ovaries and ripening fruits enclosed within corollas were higher than when corollas were removed or in the ambient conditions on sunny days. However, the increased temperature resulting from the presence of corollas was higher during fruiting than during flowering (8.0 °C *vs*. 2.0 °C; Fig. 2). Rain and clouds cancelled out such temperature differences and night-time temperature differed little between treatments during both flowering and early fruiting (Fig. 2).

#### UV-B/C radiation

**Figure 3:**
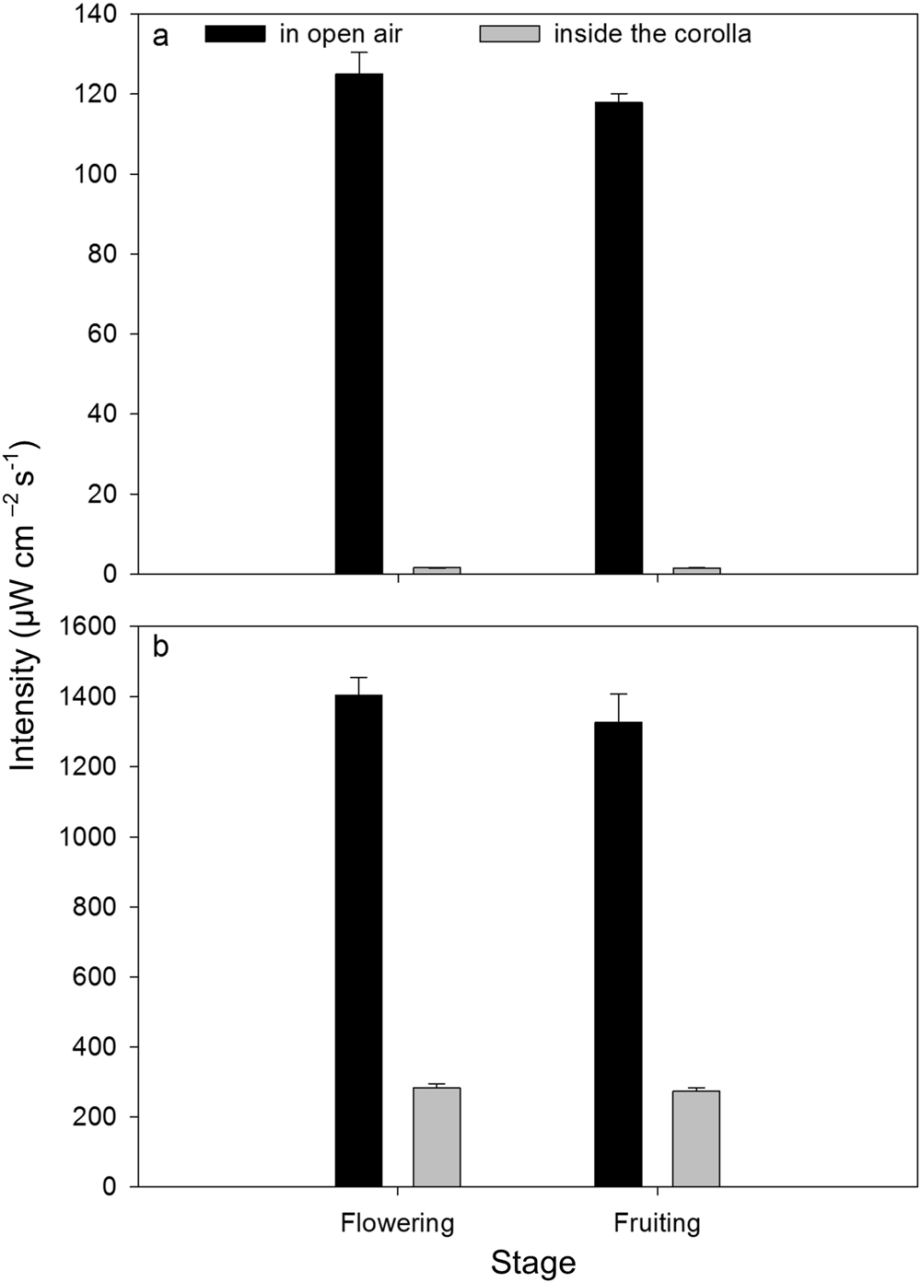
Intensity (mean ± SE) of UV-B radiation (**a**) and UV-C radiation (**b**) in open air and inside corolla during flowering and fruiting (n = 10). Analysis of these data is presented in Table 1.

There was no significant difference in intensities of either UV-B or UV-C radiation between the flowering and fruiting stages (Fig. 3; Table 1). Corollas significantly reduced the intensities of both UV-B and UV-C radiation reaching ovaries and fruits (Fig. 3), and this effect was unaffected by developmental stage (Table 1).

**Table 1:** Two-way ANOVA examining the effects of corolla treatment (inside and outside corolla) and developmental stage (flowering and fruiting) on intensities of UV-B/C radiation.

Source	UV-B			UV-C		
	*df*	*F*	*P*	*df*	*F*	*P*
Stage	1, 36	1.55	0.22	1, 36	0.80	0.52
Treatment	1, 36	1699.24	<0.001	1, 36	507.27	<0.001
Stage × Treatment	1, 36	1.49	0.23	1, 36	0.53	0.47

### Effects of corolla retention on fecundity and progeny quality

**Figure 4:**
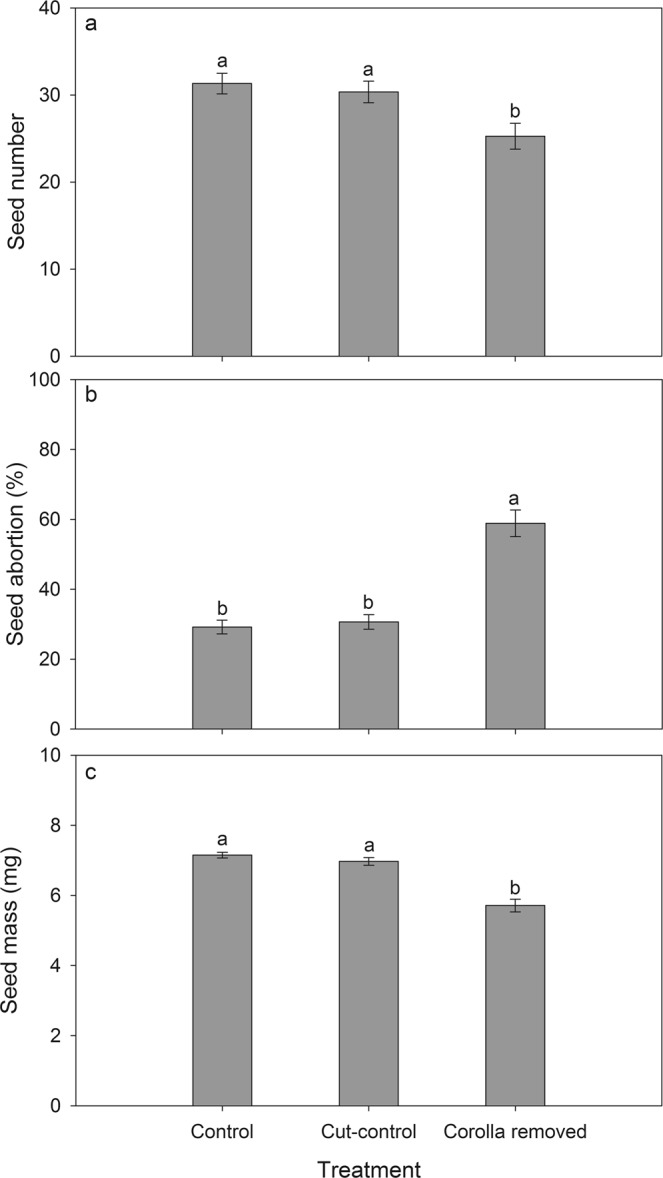
Effects of corolla treatment on seed number per fruit (**a**), seed abortion rate (**b**) and mass per seed (**c**) of *Fritillaria delavayi* (n = 20). Different letters denote significant differences at *P* < 0.05. Control: corolla was left intact; Cut-control: all tepals on the corollas were trimmed *c*. 1 mm in from their edge after flowering; Corolla removed: corollas were removed after flowering.

Seed number per fruit was significantly affected by corolla treatment (*F*_2,57_ = 6.21, *P* < 0.01; data log transformed). Seed numbers per fruit from intact plants (31.3 ± 1.2) and plants with trimmed-control corollas (30.4 ± 1.3) were not different from each other but were both significantly higher than from plants with the corolla removed (25.3 ± 1.5; Fig. 4a). Seed abortion was also significantly affected by corolla treatment (*F*_2,57_ = 36.23, *P* < 0.001; data square root transformed). The proportions of aborted seeds in intact plants (29.2% ± 2.0) and in plants with trimmed-control corollas (30.6% ± 2.1) were not different from each other but were both significantly lower than in plants with the corollas removed (58.9% ± 3.8; Fig. 4b). We also found evidence that seed mass differed significantly between treatments (*F*_2,57_ = 34.82.5, *P* < 0.001; data box-cox transformed). The mass of seeds from intact plants (7.2 ± 0.08 mg) was significantly higher than that from plants with corollas removed (5.7 ± 0.2 mg), but not different from plants with the trimmed-control treatment (7.0 ± 0.1 mg; Fig. 4c).

### Potential costs of corolla during fruiting

There was no significant variation in petal size between flowering and early fruiting (*t*_38_ = 1.74, *P* = 0.10; *t*_38_ = 1.34, *P* = 0.20 for tepal length and tepal width, respectively). The tepals were 3.60 ± 0.09 cm long and 1.48 ± 0.04 cm wide during flowering, and 3.58 ± 0.09 cm long and 1.50 ± 0.04 cm wide during early fruiting. Similarly, corolla mass did not change after flowering (*t*_38_ = 1.68, *P* = 0.10; flowering: 75.36 ± 1.2 mg; early fruiting: 72.44 ± 1.3 mg). The respiration rate of the corolla during fruiting (0.36 ± 0.07 μmol m^−2^ s^−1^) was significantly lower than during flowering (1.03 ± 0.11 μmol m^−2^ s^−1^; *t*_14_ = −4.93, *P* < 0.001). Similarly, the transpiration rate of the corolla during fruiting (0.18 ± 0.05 mmol m^−2^ s^−1^) was significantly lower than during flowering (0.42 ± 0.05 mmol m^−2^ s^−1^; *t*_14_ = −3.26, *P* < 0.01). These results suggest that the costs of respiration and transpiration for the corollas during the period of seed development were significantly reduced compared with those during flowering.

## Discussion

Adverse environmental conditions (e.g., low temperatures and high levels of UV radiation) in alpine zones have been found to constrain reproductive processes, including seed development^8,29,30^. As a result of adaptation to various abiotic stresses, plants in such habitats have developed a variety of morphological and physiological characteristics^31^. In our study, we demonstrate that corolla removal after flowering has a significantly negative effect on seed development, adversely affecting seed set, abortion rate, and seed mass, suggesting that corolla retention in *F. delavayi* facilitates the development of fertilized ovules during fruiting. Furthermore, we were able to rule out the possibility that the experimental removal of corollas may have caused damage, thereby negatively influencing seed production and seed quality, because there was no difference in reproductive output between the trimmed-control treatment and the control treatment.

Cell division and cell expansion, two important physiological activities in the process of seed development, are especially sensitive to low temperature^32,33^. Furthermore, the rate of carbon transfer from leaves and storage structures to the ovaries depends to a large extent on the temperature^34^. Therefore, it has even been suggested that reproductive output is limited by meristem activity rather than photosynthesis in cold environments^35,36^. Our results show that the persistent corollas of *F. delavayi* can increase the interior temperature of fruits by as much as 8 °C compared with the ambient air temperature on sunny days, and this may provide a favorable thermal environment for ripening seeds, thus promoting seed development. However, unlike the results reported by Seymour *et al*.^13^, that *Philodendron solimoesense* increases floral temperature by self-heating, there was no difference in temperature at night or during rain between fruits concealed by corollas and the ambient air. The temperature increase by corolla retention was only apparent on sunny days, indicating that the higher temperature depends on solar-energy absorption, similar to that reported for *Ranunculus glacialis*, *Oncocyclus* irises and *Rheum nobile*^2,8,26^. This was also indicated by the fact that gray corollas increased interior temperature during fruiting more than bright yellow corollas during flowering, probably because darker colors absorb more solar radiation than bright colors^2,7,26^. In addition, closed corollas of *F. delavayi* are able to prevent convection between the interior and the surrounding air during fruiting, functioning as an insulator and thus improving the heat storage capacity, similar to the situation described by Yang and Sun^7^ for the bracts of *Saussurea velutina*. Thus, the heat-storage function of closed corollas contributes to the interior temperature decreasing more slowly than ambient temperature when rain and overcast skies cause solar radiation to drop abruptly. A similar situation was reported in *R. nobile* from the same region, where the tightly overlapping bracts had a positive effect on heat retention^8^. Obviously, our results suggest that the corolla retention during fruiting in *F. delavayi* acts both as a heat absorber and as a buffer against rapid fluctuations in temperature.

Numerous studies have shown that intensive UV-B/C radiation can result in biological damage to nucleic acids and alteration in protein content and enzyme activity, particularly in the early stages of seed development^37–39^. In addition, intense UV-B/C radiation has been found to be deleterious to the growth of the pollen tube^40,41^, which is especially crucial for subsequent seed production. The UV-B/C levels in the alpine areas of the Hengduan Mountain region are much higher than in many low-altitude locations, and seed ripening in *F. delavayi* occurs between July and early September, when the UV radiation intensity tends to be highest in this region^42^. As Zhang *et al*.^28^ reported for the corollas or perianths of many alpine plant species, the corollas of *F. delavayi* are able to screen UV radiation efficiently; furthermore, this blocking effect was similar between flowering and fruiting, with *c*. 98 and 80% of UV-B and UV-C radiation, respectively, being excluded by the corollas. Thus, the persistent corollas of *F. delavayi* may protect growing pollen tubes and ripening seeds from damaging caused by intense UV radiation at high elevations in the alpine areas of the Hengduan Mountain region.

It is generally accepted that, with a fixed amount of resources, increased expenditure of resources on floral structures (e.g., perianths or corollas) necessarily entails reduced allocation to seed production^43,44^. For example, production and maintenance of perianths significantly reduced seed mass and the total biomass allocated to seed production in *Nigella sativa*^44^. However, we found no significant differences in either corolla mass or size between the flowering and early fruiting stage, indicating that corollas do not grow any more after flowering. Thus, the corollas originally devoted to pollinator attraction are just “re-used” during fruiting without further investment in construction cost. Corolla retention may incur maintenance costs after anthesis^21^; however, compared with corollas at the flowering stage, both respiration rate and transpiration rate of corollas at the early fruiting stage were significantly lower. Thus, a slight additional resource investment for corolla retention after anthesis contributes substantially to increasing seed production in *F. delavayi*, similar to the way that persistent bracts contribute to seed development in *R. nobile* by increasing the temperature and screening UV radiation for ripening seeds^8^.

It is worth noting that, under the ecological context hypothesis, the adaptive value of corolla persistence may be influenced by the composition and abundance of organisms with which a species interacts^15^. It is possible that persistent corollas of *F. delavayi* reduce predispersal seed predation by preventing predators reaching the seeds^16^, or they may have detrimental effects on plant fitness by providing fruit herbivores with a refuge from enemies, resulting in increased seed predation^15,19^. However, we did not find any evidence of predation in the fruits with corollas removed or with corollas intact in either 2015 or 2016 (Gao Y *et al*. unpublished data), indicating that corolla persistent may not be an antiherbivore adaptation, or result in a frugivory cost, at least at the two sites studied.

In conclusion, our study demonstrated significant reproductive benefits of corolla retention during fruiting of *F. delavayi*. Our results show that the temperature of developing fruits is increased and the intensity of UV-B/C radiation reaching developing fruits is decreased due to corolla retention, and seed production by flowers with corollas removed after flowering is significantly reduced compared with intact flowers. These findings indicate that the persistent corollas of *F. delavayi* provide an important protective role for the fertilized ovules during fruiting. Compared with this protective effect, the resource cost incurred by corolla retention during fruiting is very modest. Consequently, our study suggests that corolla retention during fruiting in *F. delavayi* is an adaptive strategy, enhancing reproductive output in the harsh conditions at high elevations.

## Materials and Methods

### Species description and study sites

*Fritillaria delavayi* Franch (Liliaceae) is a perennial medicinal herb growing to a height of 35 cm, with a relative large bulb, founding at elevations between 3800 and 4700 m, and inhabiting mostly open scree in the Hengduan Mountains, southwestern China^45^. The plant flowers between June and July, with each plant producing a solitary pendulous, bell-shaped, terminal flower. *F. delavayi* is self-compatible, and autonomous selfing may occur, but the plant depends mostly on bumblebees for pollination^46^. After anthesis, the corolla is maintained and encloses the fruit. Tepals change color from bright yellow to gray (Fig. 1). Seeds mature between late August and early September.

The field experiments were conducted between June and September 2016, at two sites: Yongjiongyi (28°25′N, 99°55′E, a.s.l. 4657 m), in Shangri-la County, Yunnan Province, SW China, and Xinduqiao (30°05′N, 101°48′E, a.s.l. 4200 m), in Kangding County, Sichuan Province, SW China. The study populations are located on open alpine scree, which has a summer monsoonal climate characterized by cold rain interrupted by brief periods of intense solar radiation^47^.

### Effects of corolla retention on interior microenvironment

#### Temperature

Six plants were randomly selected during both flowering and early fruiting to assess the effect of corolla retention on the temperature of developing ovules^7,8^. The corollas of three of these plants were removed carefully with forceps (treatment: corolla removed) and those of the other three plants were left intact. Temperatures of the ovaries were measured in both flowers with corollas removed and intact individuals using 4-channel thermocouple data loggers (Center 309, data logger thermometer, Center Technology Corp., Taiwan, China), each equipped with four alloy needle-type sensor probes (1–3 mm in diameter and with an active tip length of 5 mm); data were collected between 3 and 6 July 2016, and all days included sunny and rainy periods. The air temperature (*c*. 20 cm above the ground) was measured using an integrated thermistor (1400-104 air temperature sensors; LI-COR, Lincoln, NE). The data loggers were programmed to sample temperature every 5 min. The measurements during early fruiting were conducted between 21 and 24 July 2016, in the same way as during flowering.

#### UV-B/C radiation

To test the effects of corollas on interior intensities of UV-B/C radiation, five plants were selected randomly each day during both the flowering and the early fruiting stage. Following the method of Yang and Sun^7^ and Song *et al*. ^8^, intensities of UV-B/C radiation inside corollas were measured in the native habitat using UV-radiometers (Photoelectric Instrument Factory of Beijing Normal University) at 14:00 h on 7 and 8 July, and 25 and 26 July 2016 during flowering and early fruiting, respectively. Similarly, the intensities of UV-B/C radiation in open air were measured. In total, 40 measurements for UV-B and 40 measurements for UV-C radiation were conducted.

### Effects of corolla retention on fecundity and progeny quality

To test the effect of corolla retention on fecundity and progeny quality, we randomly selected 60 flowering plants with similar sizes and supplementarily pollinated them with outcrossed pollen grains to exclude the effects of pollen limitation on seed production. These plants were different from those used for the temperature and UV measurements. Twenty each of the selected plants were randomly assigned to one of the following three groups: (1) control: corollas were left intact; (2) corollas removed after flowering: once stigmas were no longer receptive, the corollas were carefully removed with forceps; (3) control for cut corollas: once stigmas were no longer receptive, all tepals on the corollas were cut *c*. 1 mm in along their edge as a control for damage but maintaining their size as far as possible. When fruits were ripe (in early September), all fruits were collected and taken to the laboratory to determine seed number, seed abortion rate and seed mass. Seeds that were not fully expanded and were empty were classified as aborted. For seed mass, 10 seeds selected randomly from each plant were measured individually and each plant was treated as a replicate.

### Potential costs of corolla during fruiting

To assess the carbon allocation during flowering and early fruiting, 20 flowering plants were selected randomly and marked. Length and width of each tepal were measured for each flower at the flowering and early fruiting stage. In addition, 40 flowering plants were selected randomly before flowering to measure the mass of corollas. The plants were separated into two groups. In the first group, the corolla of each plant was collected and brought back to the laboratory during flowering. For the second group, the corollas were collected at early fruiting. All samples were oven dried at 75 °C for 48 h and then weighed.

To determine the respiration and transpiration costs of corollas, eight plants were selected randomly during flowering and eight during early fruiting to measure the rates of respiration and transpiration of corollas using a portable photosynthesis measurement system with a fluorescence chamber head (LI-6400-40, Li-Cor, Lincoln, NE, USA), with CO_2_ concentration at *c*. 380 μmol mol^−1^ and quantum flux at *c*. 2000 μmol m^−2^ s^−1^, representing sunny condition. Measurements were made on two tepals for each corolla and means were calculated for each corolla before analysis.

### Data analysis

One-way ANOVA was used to test the effect of corolla retention on seed number, seed abortion and seed mass. In order to test the effect of corolla and developmental stage on intensity of UV-B/C radiation, two-way ANOVA was used. A paired *t* test was performed to test the difference in tepal size and an independent-sample *t* test was performed to test the difference in corolla mass, respiration and transpiration rate between flowering and early fruiting, respectively. Multiple comparisons of means were performed using *Tukey’s* test at the 0.05 significance level. Data were log transformed, square-root transformed or box-cox transformed as necessary to meet ANOVA assumptions. Normality was tested with normal probability plots; homogeneity of variance was tested using Levene’s test. All analyses were performed using SPSS ver. 18.0. Measurements are reported as means ± 1 SE.
